# Distinguishing task shifting from task sharing through the delegating provider’s presence or absence: proposed operational definitions

**DOI:** 10.7189/jogh.16.03034

**Published:** 2026-10-02

**Authors:** Shukanto Das, Liz Grant

**Affiliations:** Centre for Global Health, Usher Institute, University of Edinburgh, Edinburgh, UK

**Keywords:** task shifting, task sharing, operational definitions, health workforce, primary healthcare, mental health, emergency medical services

## Abstract

Task shifting and task sharing are strategies for redistributing healthcare responsibilities to optimise healthcare services. The World Health Organization (2008) framed ‘shifting’ as the delegation of tasks from higher- to lower-qualified workers, while subsequent scholarship characterised ‘sharing’ as collaborative, team-based redistribution. However, the two approaches are often used interchangeably in literature and by practitioners. Drawing on interviews and observations across 16 Indian organisations working in primary healthcare, mental health, and emergency medical services, we identified two types of operational ambiguities: organisations simultaneously employed both strategies for different tasks within the same service and also demonstrated practices that did not match their design intent or initial appearances. Based on these, we reiterate the importance of classifying task redistribution (Shifting OR Sharing) at individual task levels, consistent with the task-specific wording of conventional definitions, and additionally propose a new operational criterion: whether the delegating provider is functionally present or absent during task execution. We suggest revised working definitions incorporating this criterion as a hypothesis for prospective testing, and invite the global health community to validate these for clearer implementation, evaluation, and policy.

Shortages of healthcare providers are estimated to reach 11–18 million globally by 2030 [1–3]. Task shifting and task sharing (TS/S) are two strategies to close gaps across multiple domains [4–6]. The World Health Organization (WHO) 2008 guidelines, with the President’s Emergency Plan for AIDS Relief and the Joint United Nations Programme on HIV/AIDS, defined task shifting as ‘the rational redistribution of tasks among health workforce teams’, whereby ‘specific tasks are moved, where appropriate, from highly qualified health workers to health workers with shorter training and fewer qualifications’ [7]. The European Commission’s Expert Panel on Effective Ways of Investing in Health similarly saw it as transferring responsibilities from more to less-expert workers [8]. Task sharing differs as a concept in that among teams of providers, ‘tasks are not taken away from one cadre and given to another, but rather additional cadres are given the capacity to take on identified tasks’ [9,10]. Indeed, ‘sharing’ conveys that care is shared within a team, not transferred from one cadre to another [11,12]. The COATS framework [13] consolidated these nuances: ‘shifting’ focuses on delegation, while ‘sharing’ concerns collaboration between providers with different skill sets.

Yet the terms are used interchangeably, often to describe transfers down a professional hierarchy. Our bibliographic analysis of 2072 TS/S publications (1970–2022) found 66% used ‘task shifting’, 24% ‘task sharing’, and many used both without operational differentiation [4]. This is not mere semantics; it affects how TS/S is planned, done, and analysed. We therefore report how this overlap manifests in practice; underscore the importance of task-level classifications consistent with the wording of existing definitions; and propose one new operational criterion to reduce ambiguities.

## FIELDWORK IN INDIA

A study to validate and refine a new TS/S-specific implementation framework, the SHIFT-SHARE [14,15], by examining how observed practice aligned with its six stages [16], was conducted in India between November 2023 and September 2024 [17]. This work comprised 30 semi-structured interviews, eight group discussions and seven participant observations across 16 healthcare organisations; resultant framework updates from which are reported separately [15]. Each organisation constituted one case: a unit with distinct leadership, defined services, identifiable task redistribution between cadres of different training levels, and sufficient data for analysis. Participants were recruited purposively, supplemented by convenience and snowball sampling, against predefined inclusion and exclusion criteria [17]. The 16 cases spanned three domains, namely primary healthcare (five cases), mental health (MH, four) [16], and emergency medical services (seven), across eight Indian states. They ranged from non-governmental organisations training village-based community health workers (CHWs) to government primary care facilities, nurse-led primary health centres (PHCs) in tribal areas, corporate well-being programmes, private ambulance services, and hospital emergency departments. The cadres involved included CHWs, accredited social health activists (ASHAs), nurses, paramedics, mid-level health workers, peer volunteers, and doctors of India’s AYUSH system of alternative medicine. Of the seven observations, two covered skill-building workshops and five covered task execution, lasting between one hour and half a day each (Table S1 in the **Online Supplementary Document**).

We used a hybrid deductive-inductive approach to analyse findings [18,19]. The first author, SD, coded data, with interpretations tested *via* member checking, triangulation across the three data collection modalities, and review with the senior author (LG). We tracked theme recurrence through structured content summary notes, with major ones recurring by around the eighteenth interview. We appraised sample adequacy through information power [20].

This paper extends that work in a direction the parent study did not set out to explore: the classification ambiguities reported here were not an *a priori* focus. They emerged inductively, after all 16 cases had been constituted in the parent study, when service-level classification with classical definitions kept failing (Table S2 in the **Online Supplementary Document**), and participants themselves used the terms interchangeably. We draw on the full set of cases to discuss definitional clarity issues next.

## CONCEPTUAL OVERLAP IN PRACTICE

Classical definitions operate at task levels: the WHO explicitly refers to ‘specific tasks’ being moved [7]; though much policy and implementation discourse applies the terms at programme or systems level, so insisting on task-level granularity partly reinterprets prevailing usage even where it restates definitional wording. In practice, people label entire services or organisations as either ‘shifting’ or ‘sharing’. Classifying each of our 16 cases this way using classical definitions produced ambiguities; many participants were themselves unsure which term described their activities, using the two interchangeably, echoing the inconsistency in literature [4]. We identified two forms.

### Type A: simultaneous use of both strategies within a single service

Simultaneous use means that within one service some tasks meet the criteria for ‘shifting’ (delegation to a less-qualified cadre) while others meet those for ‘sharing’ (collaborative redistribution). A non-profit in Kerala runs community MH services for marginalised fishing communities through 80 clinics staffed by clinical psychologists and CHWs: the CHWs made independent, unsupervised home visits for medication adherence monitoring (delegation), yet jointly delivered behavioural components of therapy alongside psychologists in clinic sessions (collaboration). Another non-profit-run PHC in rural Chhattisgarh showed the same pattern: locally recruited mid-level health workers (tribal youth with basic schooling and no formal health qualifications) independently handled blood draws and vital signs, while nurses ran outpatient consultations, yet also assisted those nurses under direct supervision in the busy pharmacy and laboratory. A government PHC in Gujarat showed a related pattern: nurses independently diagnosed malaria with rapid diagnostic kits when the physician was absent, but delivered health education and managed national digital-programme data entry collaboratively with ASHAs and female health workers. In all cases, the same staff operated under both models depending on the task, so neither service could be characterised as ‘shifting’ or ‘sharing’ alone.

This has practical consequences. For instance, classified as ‘sharing’, the Kerala service’s independent home visits get oversight assuming professional co-presence; classified as ‘shifting’, its collaborative therapy sessions get supervision designed for delegated work. Either label misaligns with part of the service. Re-examined task by task, every Type A ambiguity was resolved. At the Kerala MH service, home visits become ‘shifting’; joint therapy sessions are ‘sharing’. At the Chhattisgarh PHC, independent vitals measurement is ‘shifting’; supervised pharmacy assistance is ‘sharing’. At the Gujarat PHC, malaria diagnosis is ‘shifting’; collaborative health education is ‘sharing’. These are simply different tasks with different operational characteristics coexisting in one service. Consistent task-level application, therefore, would prevent the false dichotomies that arise from service- or organisation-wide labelling.

### Type B: practice does not match design intent or initial appearance

The second form is a misalignment between the classification a programme’s stated design or structure implies and what observation reveals. This is distinct from Type A because, even at task level, what a reasonable first assessment would conclude does not hold under closer examination. This matters because programmes build supervision, legal liability, and quality monitoring around their assumed model. When reality diverges, accountability gaps open: an independent provider may lack the oversight a supposedly collaborative role assumes, and a collaborating provider may lack the autonomy a delegated role was designed to provide.

A private multispecialty hospital in Gujarat hired a Bachelor of Ayurvedic Medicine and Surgery-trained doctor, within India’s AYUSH system [21], for its emergency department, specifically to cut consultant workload by independently assessing patients, taking histories, and managing routine cases; a role designed as shifting. Observation and interviews showed otherwise: the AYUSH doctor consistently deferred clinical decisions to the allopathic consultant, ordered no investigations until the consultant was present, never kept patients under their own care, and recorded all documentation under the consultant’s credentials. A role designed as ‘shifting’ functioned as ‘sharing’. A construction and technology conglomerate headquartered in Mumbai (over 200,000 staff, with full-time professional counsellors on payroll) showed the reverse. It designed an MH programme as task sharing: volunteer employees were trained and certified over six months, with psychiatrists and psychologists as peer MH first aiders complementing the existing counselling services. But the very barrier the programme was created to address (stigma and fear of career consequences) kept employees from approaching counsellors directly, so volunteers became the primary, often sole, contact point, independently identifying concerns, giving initial support, and managing colleagues’ distress without professional involvement. A programme designed as ‘sharing’ operated as ‘shifting’.

Both types of ambiguity point to one challenge: current definitions, though conceptually sound, lack the operational specificity for working classifications at points of care. Without observable criteria, practitioners mischaracterise their own processes, and researchers cannot draw meaningful cross-context comparisons. Task-level classification resolves Type A by recognising that different tasks in one service can follow different strategies, but not Type B, where even task-level claims diverge from observation. For this, a criterion grounded in observable practice is needed.

## PROPOSING THE PRESENCE/ABSENCE CRITERION

What operationally distinguishes a shifted task from a shared one? Current definitions frame the distinction as delegation *vs* collaboration [13], but specify no visible indicators. Across our cases, one characteristic gave a reliable basis for classification: whether the delegating (higher-qualified) provider was physically or functionally present during task execution. In shifting, the receiving cadre works independently or in the delegating provider’s absence, even where periodic supervision or remote monitoring is maintained. In sharing, both cadres are functionally present. We define functional presence by an explicit threshold: the delegating provider is engaged in synchronous, bidirectional clinical decision-making during the execution of the task itself, with the capacity to alter the task while under way. Availability, whether before, after, or on request, does not constitute presence.

Supervision arrangements observed across our cases fall on a gradient: (i) on-site co-presence with active collaboration; (ii) remote but real-time clinical engagement, such as continuous telephone guidance with structured read-back; (iii) on-demand availability; (iv) periodic scheduled supervision, such as weekly visits or calls; and (v) retrospective or administrative review. Under the threshold above, arrangements (i)–(ii) constitute functional presence; (iii)–(v) constitute absence (Table S3 in the **Online Supplementary Document****)**. This connects to wider literatures: the sociology of professions treats jurisdiction over tasks (who may perform them, under whose oversight) as a core criterion of professional negotiation [22]; distributed cognition locates clinical decision-making in interactions between actors rather than individuals [23]; and interprofessional-collaboration research draws a similar line between co-located teamwork and delegated autonomy [24]. Our contribution is specific: an observable, task-level marker of where an arrangement sits.

Applied to Type B cases, the criterion classifies by observation, not intent. At the Gujarat hospital, the design described independent practice, but the delegating provider was not absent – the consultant was present and deferred to – so the criterion classifies ‘sharing’, catching the design–reality mismatch. In the Mumbai programme, the design described collaboration, but the delegating provider was not present – counsellors were absent from volunteers’ interactions – so it classifies ‘shifting’, again catching the divergence. The criterion also confirms other intuitive classifications in unambiguous cases. In rural Rajasthan, *e.g.* a non-profit’s six nurse-led PHCs serve 90,000 mainly tribal people where physician-dependent PHCs had failed: local nurses independently manage consultations, acute and maternal care, pharmacy, inventory, and CHW supervision; doctors visit weekly and are telephone-available but routinely absent. The criterion confirms ‘shifting’: the delegating provider is absent during task execution. Similarly, at one occupational health centre in Gujarat, a lone paramedic with a nursing diploma independently handles consultations, trauma assessment, suturing, and cardiopulmonary resuscitation for occupational injuries, with no doctor on site (though, for compliance, documentation is kept under a visiting doctor’s name) – again, ‘shifting’. This case also illustrates a distinction the criterion depends on: clinical autonomy, administrative supervision, legal responsibility, and real-time collaboration are separable dimensions that do not always align. Here, *e.g.* practical autonomy sits with the paramedic, legal accountability with an absent physician. The criterion classifies only the operational dimension, *i.e.* who is engaged in the task as it happens, deliberately bracketing legal and administrative arrangements, which existing definitions also leave unresolved.

Conversely, in another example of a state government directorate in Delhi, co-locating AYUSH and allopathic units so that patients access both systems under one roof, doctors keep separate consultation areas but manage complementary caseloads *via* cross-referrals (chronic conditions to AYUSH, acute ones to allopathic physicians), both present within shared infrastructure. The criterion confirms ‘sharing’: both providers are functionally present. Another case of a national private-based road and air ambulance service showed that functional presence needs no co-location: paramedics trained in advanced procedures (electrocardiogram interpretation, cardiac protocols, medication administration) perform them during critical transports while consulting call-centre doctors in real time: the paramedic assessing and executing, the doctor confirming medication routes and dosages *via* structured read-back. Both cadres decide at the same point of care, classifying this as ‘sharing’ as the delegating provider is functionally present, even though physically remote.

From these observations, we propose working operational additions to existing definitions (**Box 1**). These do not replace them but add an observable characteristic supporting consistent classifications. The WHO framing of ‘shifting’ as rational redistribution [7] and the COATS framework’s distinction between delegation and collaboration [13] remain valid. Our suggestion operationalises what each looks like at the point of care.

Box 1 **Proposed working operational additions to definitions of task shifting and task sharing**

**Task Shifting**
**Existing definition**: The rational redistribution of tasks among health workforce teams, whereby specific tasks are moved, where appropriate, from highly qualified health workers to health workers with shorter training and fewer qualifications.
**Proposed addition**: ‘*...who perform these tasks while the delegating provider is functionally absent, taking no part in real-time clinical decision-making during task, even where periodic supervision, on-demand availability, or retrospective review is maintained.*’
**Task Sharing**
**Existing definition**: A team-based approach where care is provided by a mix of health workers collaborating and expanding on roles
**Proposed addition**: ‘*...with each provider functionally present and jointly engaged in clinical decision-making within the same episode of care, whether contributing simultaneously or in sequence.*’

## LIMITATIONS AND IMPLICATIONS

Our observations come from one country. India’s workforce configurations, including AYUSH doctors and ASHAs, and regulatory environment are not representative of all systems elsewhere; community health programmes in sub-Saharan Africa or nurse-prescribing models in southern Africa may pose classification challenges our cases did not capture. Our recommendation is therefore scoped accordingly. The criterion is a testable working proposal, not a validated global standard. We advance the criterion as a hypothesis generated from secondary analysis of our fieldwork, not as empirically validated operational definitions. Its reliability and validity therefore need further prospective testing.

All classifications were made by the first author and reviewed with the senior author. We did not conduct a formal inter-rater reliability testing across independent observers. The threshold and typology above provide the operational guidance such testing would need, but borderline arrangements, for instance, intermittent synchronous contact, will still demand judgment and, likely, domain-specific guidance.

Our findings across domains are consistent with a gap in the definitions themselves, supported by the interchangeable usage documented internationally [4]. Some contributing explanations cannot be overlooked, such as contextual variability, uneven implementation fidelity, worker shortages forcing role adaptations, and local interpretations of hierarchy, which may each amplify ambiguities observed. We acknowledge that role redistribution can exist on a continuum of provider interdependence, meaning that the terms may not always be ontologically discrete. However, our criterion draws a line across that continuum at the task level, and we draw it deliberately: the decisions that depend on classification, such as supervision structures, evaluation designs, and accountability arrangements, are yes-or-no decisions in practice. Clinical practice does the same when it sets thresholds on continuous measures because a decision has to be made.

We suggest a staged validation pathway with inter-rater reliability testing, with independent observers applying the threshold and typology to standardised vignettes and observations; prospective observational validation beyond India, sampling supervision architectures our cases did not contain; implementation audits comparing declared classifications with criterion-based observation, testing whether the criterion detects design–practice mismatches (our Type B) at scale; and comparative studies of whether criterion-consistent classification predicts outcomes that programme-level labels do not. For instance, whether supervision structures matched to observed presence or absence associate with safety and quality indicators. Success along this pathway is what would justify adoption of our definitions.

Furthermore, we note that the terminology is also politically and legally charged. ‘Sharing’ can be preferred precisely because it softens implications of hierarchy and substitution [12], while professional bodies may resist classifications that make delegation visible, seeing it as jurisdictional encroachment. An observable criterion cuts both ways: it constrains euphemistic labelling but may equally surface politically and legally sensitive classifications that programmes had reasons to leave ambiguous, for instance, where documentation and liability depend on nominal supervision by a functionally absent provider. We regard this transparency as a feature for governance, though implementation will need sensitivity to the professional politics involved.

We invite the global health community to test our criterion, which we state as a hypothesis generated in the Indian context, requiring validation across other workforce configurations and regulatory environments, alongside the task-level classification that definitions already suggest. Testing and adopting it could strengthen evaluation, letting stakeholders check whether supervision structures match the actual extent of provider independence for each redistributed task; improve cross-context comparability; and support clearer policy guidance. Frameworks like the SHIFT-SHARE [14,15] could incorporate this parameter. As TS/S expands, ensuring foundational terminology resolves rather than extends conceptual overlap is key for the communities these strategies serve.

## Additional material


Online Supplementary Document

## Data Availability

**Data availability:** All relevant data supporting the findings are within the paper and its **Online Supplementary Document**.
