# Supplementary material for: Distinguishing task shifting from task sharing through the delegating provider’s presence or absence: proposed operational definitions
Source: J Glob Health. 2026 Oct 2;16:03034. doi: 10.7189/jogh.16.03034 (PMC13631933; doi:10.7189/jogh.16.03034)
Supplement: Supplementary file 1 [file jogh-16-03034-s001.pdf]

**Supplement to: Das S, Grant L. Distinguishing task shifting from task sharing through the delegating provider's presence or absence: proposed operational definitions. J Glob Health. 2026;16:03034.**

**Table S1: The 16 cases: settings, cadres, tasks redistributed, and data sources**

SSI = semi-structured interview; PO = participant observation; FGD = focus group discussion. All cases were constructed from SSIs and POs, supplemented by FGDs where indicated.

| Case | Domain | Setting and cadres | Tasks redistributed | Data sources |
|---|---|---|---|---|
| 01 | Primary health care | NGO community health programme, rural; CHWs taking on work of doctors and nurses | Screening, health education, referrals, and water-sanitation-hygiene activities, from doctors/nurses to CHWs | SSIs with 4 participants; PO (CHW-led screening and education); 3 members represented in FGDs* |
| 02 | Primary health care | Government PHC, Gujarat; medical officers, nurses, ASHAs, and female health workers | Malaria rapid-test diagnosis, TB identification, and antenatal care to nurses; outreach, health education, and digital data entry with ASHAs and FHWs | SSIs with 3 participants; PO (ASHA- and FHW-led outreach) |
| 03 | Primary health care | NGO nurse-led primary care clinics, rural/tribal Rajasthan (six clinics, ~90,000 population) | Outpatient consultations, acute and maternal care, pharmacy and inventory management, and CHW supervision, from doctors to nurses | SSIs with 6 participants; PO (nurse-managed outpatient care without an on-site doctor) |
| 04 | Primary health care | NGO rural clinics, Chhattisgarh; nurses and locally recruited mid-level health workers (MLHWs) | Outpatient consultations to nurses; blood draws and vital signs to MLHWs, who also assist nurses in pharmacy and laboratory | SSIs with 2 participants |
| 05 | Primary health care | State government directorate, Delhi; co-located AYUSH and allopathic doctors, non-clinical staff | Complementary caseload management via cross-referrals between AYUSH and allopathic doctors; pharmacy dispensing by non-clinical staff | SSI with 1 participant |
| 06 | MH | NGO, rural Gujarat; CHWs taking on work of psychiatrists | MH screening, counselling, medication management, and rehabilitation, from psychiatrists to CHWs | SSIs with 4 participants (including a group interview with |

| | | | | |
|---|---|---|---|---|
| | | | | trustees); PO (home-based MH follow-up) |
| 07 | MH | NGO, Kerala; ~80 community MH clinics for marginalised fishing communities; clinical psychologists and CHWs | Independent home visits for medication adherence monitoring by CHWs; behavioural components of therapy delivered jointly with psychologists | SSIs with 3 participants |
| 08 | MH | Corporate peer mental-health programme, Mumbai-headquartered conglomerate (>200,000 staff) | Early identification, initial psychological support, and referral of distressed colleagues, from professional counsellors to trained peer volunteers | SSI with 1 participant |
| 09 | MH | Border security force programme training nurses as peer counsellors | Peer counselling, from MH professionals to nurses | SSI with 1 participant |
| 10 | EMS | ASHA paediatric first-responder programme | Paediatric emergency first response (CPR, airway and choking management), from doctors to ASHAs | SSI with 1 participant |
| 11 | EMS | Occupational health centres across industrial sites, Gujarat and Rajasthan; single-paramedic port centre | Outpatient consultations, trauma assessment, suturing, and cardiopulmonary resuscitation, from doctors to a paramedic | SSI with 2 participants |
| 12 | EMS | Private road and air ambulance service, national | Electrocardiogram interpretation, emergency cardiac protocols, and medication administration during transport, to paramedics guided in real time by call-centre doctors | SSIs with 5 participants; PO (training session preparing ambulance drivers to assist paramedics); 1 member represented in FGDs* |
| 13 | EMS | State trauma centre, New Delhi; dedicated trauma nursing cadre | Triage, vital-sign assessment, blood sampling, and equipment setup, from residents to trauma nurse coordinators | SSI with 1 participant |
| 14 | EMS | Programme transferring specialist emergency | Specialist emergency care for high-burden, time- | SSI with 1 participant; 6 members represented in FGDs* |

| | | | | |
|---|---|---|---|---|
| | | care to MBBS doctors at PHC level | sensitive conditions, from specialists to MBBS doctors | |
| 15 | EMS | Trauma-care training programme for AYUSH doctors | Pre-hospital trauma and emergency response, from allopathic to AYUSH doctors via structured training | SSIs with 4 participants; PO (one-day trauma-care workshop for AYUSH doctors); 1 member represented in FGDs* |
| 16 | EMS | Private multispecialty hospital emergency room, Gujarat; AYUSH doctor alongside allopathic consultants | Emergency-room patient assessment, history-taking, and routine case management, from consultants to an AYUSH doctor | SSIs with 6 participants; PO (AYUSH-staffed emergency care) |

*FGD representation counts members of a case among the eight focus group discussions of the parent study, which gathered stakeholder feedback on the SHIFT-SHARE framework rather than case-level data and drew additional participants from eight organisations outside the sixteen cases; their contribution to this paper is therefore indirect. Counts are derived from participants quoted in the parent study and may understate representation.

---

**Table SS2: Classification patterns under classical definitions and the ambiguities observed**

Type A = both strategies used for different tasks within one service.

Type B = observed practice diverged from design intent or initial appearance.

| Case | Pattern under classical definitions | Ambiguity and main-text discussion |
|---|---|---|
| 01 | Leans towards shifting | None observed; classified consistently; not discussed individually in main text |
| 02 | Leans towards shifting; both patterns present at task level | Type A; discussed in main text |
| 03 | Shifting (unambiguous) | None; discussed in main text as a confirmatory case |
| 04 | Shifting; mixed at task level | Type A; discussed in main text |
| 05 | Sharing and shifting elements | Discussed in main text as a confirmatory sharing case |
| 06 | Shifting | None observed; classified consistently; not discussed individually in main text |
| 07 | Leans towards sharing; mixed at task level | Type A; discussed in main text |

| | | |
|---|---|---|
| 08 | Designed as sharing; functioned as shifting | Type B; discussed in main text |
| 09 | Leans towards sharing | None observed; classified consistently; not discussed individually in main text |
| 10 | Shifting | None observed; classified consistently; not discussed individually in main text |
| 11 | Shifting (unambiguous) | None; discussed in main text as a confirmatory case |
| 12 | Leans towards shifting; classifies as sharing under the presence/absence criterion | Discussed in main text (remote functional presence) |
| 13 | Shifting (initially appears collaborative) | Appearance-practice divergence; not discussed individually in main text |
| 14 | Shifting | None observed; classified consistently; not discussed individually in main text |
| 15 | Leans towards shifting | None observed; classified consistently; not discussed individually in main text |
| 16 | Designed as shifting; functioned as sharing | Type B; discussed in main text |

---

**Table S3: Typology of supervision arrangements and their classification under the presence/absence criterion**

Threshold: the delegating provider is functionally present when engaged in synchronous, bidirectional clinical decision-making during the execution of the task itself, with the capacity to alter the task while it is underway. Availability (before, after, or on request) does not constitute presence.

| Level | Arrangement | Description | Case example | Classification |
|---|---|---|---|---|
| (i) | On-site co-presence with active collaboration | Both cadres physically co-located and jointly engaged in decision-making during the task | Case 05: co-located AYUSH and allopathic doctors managing complementary caseloads; Case 04: MLHWs assisting nurses under direct supervision in pharmacy/laboratory | Functional presence → task sharing |
| (ii) | Remote, real-time clinical engagement | Delegating provider physically remote but synchronously engaged: continuous guidance, decisions confirmed | Case 12: paramedics performing advanced procedures during transport with continuous | Functional presence → task sharing |

| | | | | |
|---|---|---|---|---|
| | | during the task, structured read-back | call-centre physician guidance | |
| (iii) | On-demand availability | Delegating provider reachable if needed but not engaged in the task; contact initiated only by exception | Case 03: doctors available by telephone to nurse-led clinics; Case 11: doctors at other sites reachable by the lone paramedic | Functional absence → task shifting |
| (iv) | Periodic scheduled supervision | Regular but non-synchronous oversight: scheduled visits or calls, feedback on past performance | Case 03: weekly doctor visits to nurse-led clinics | Functional absence → task shifting |
| (v) | Retrospective or administrative review | Oversight expressed only through documentation, compliance, or post-hoc review | Case 11: documentation maintained under a visiting doctor's name for compliance | Functional absence → task shifting |

Note: Borderline arrangements, for instance, intermittent synchronous contact during a task, need judgment and domain-specific guidance. Testing whether this typology supports consistent classification across raters is the first stage of the validation pathway proposed in the main text.
